# Canadian Public Safety Personnel and Occupational Stressors: How PSP Interpret Stressors on Duty

**DOI:** 10.3390/ijerph17134736

**Published:** 2020-07-01

**Authors:** Rosemary Ricciardelli, Stephen Czarnuch, R. Nicholas Carleton, James Gacek, James Shewmake

**Affiliations:** 1 Department of Sociology, Memorial University of Newfoundland, St. John’s, NL A1C 5S7, Canada; jws281@gmail.com; 2 Department of Electrical and Computer Engineering/Discipline of Emergency Medicine, Memorial University of Newfoundland, St. John’s, NL A1B 3X5, Canada; sczarnuch@mun.ca; 3 Department of Psychology, University of Regina, Regina, SK S4S 0A2, Canada; nick.carleton@uregina.ca; 4 Department of Justice Studies, University of Regina, Regina, SK S4S 0A2, Canada; james.gacek@uregina.ca

**Keywords:** public safety personnel, occupational stress, organizational stress, operational stress

## Abstract

Canadian public safety personnel (e.g., correctional workers, firefighters) experience potential stressors as a function of their occupation. Occupational stressors can include organizational (e.g., job context) and operational (e.g., job content) elements. Operational stressors (e.g., exposures to potentially psychologically traumatic events) may be inevitable, but opportunities may exist to mitigate other occupational stressors for public safety personnel. Research exploring the diverse forms of stress among public safety personnel remains sparse. In our current qualitative study we provide insights into how public safety personnel interpret occupational stressors. We use a semi-grounded thematic approach to analyze what public safety personnel reported when asked to further comment on occupational stress or their work experiences in two open-ended comment fields of an online survey. We provide a more comprehensive understanding of how public safety personnel experience occupational stress and the stressors that are unique to their occupations. Beyond known operational stressors, our respondents (*n* = 1238; *n* = 828) reported substantial difficulties with organizational (interpersonal work relationship dynamics; workload distribution, resources, and administrative obligations) and operational (vigilance, work location, interacting with the public) stressors. Some operational stressors are inevitable, but other occupational stressors can be mitigated to better support our public safety personnel.

## 1. Introduction

Relative to the general population, public safety personnel (PSP) [e.g., border services officers, public safety communications officials, correctional workers, firefighters (career and volunteer), Indigenous emergency managers, operational intelligence personnel, paramedics, police] [1,2] appear at increased risk for compromised physical [3], psychological [4], and emotional [5] well-being. The increased risk PSP experience is due, at least in part, to potentially psychologically traumatic event (PPTE) [2] exposures [6,7]. PPTEs refer to actual or threatened incidents of compromised physical, sexual, and mental well-being (e.g., acts of violence, fires, accidents resulting in fatalities, and explosions) [1,6,8,9]. Exposure to one or more PPTEs is common (i.e., 89.7%) in the general population [10]; however, most PSP report repeated exposures (i.e., more than 11 exposures over their lives) to diverse types of PPTE, with some PSP reporting more exposures than they can count [6,7]. Carleton et al. [4,11] highlighted that PSP report symptoms consistent with various mental health disorders at higher rates than expected for the general population. Consequences of compromised mental health can also include physical (e.g., digestive, cardiovascular disease) [12] and behavioural challenges (e.g., maladaptive responses like burnout, alcoholism, and suicidality) [6,13,14,15].

The impact of PPTEs can be exacerbated by stressors within the work environment [14]; as such, researchers have worked to clarify how occupational stressors, including organizational (e.g., job context such as inconsistent leadership styles) and operational (e.g., job content such as negative encounters with the public) stressors, shape PSP experiences, mental health, and well-being [16,17,18,19]. For example, Carleton and colleagues [14] found evidence that PSP report work-related stressors (e.g., staff shortages, shift work) that were associated with mental health challenges including anxiety and mood disorders, even after controlling for PPTE exposures. Yet, there is a lacuna in knowledge and research around the experiences and associated interpretations of organizational and operational stress within and across PSP groups from their perspectives. In response, we build on the work of Carleton et al. [14] by qualitatively characterizing work-related stressors as voiced by Canadian PSP in response to open-ended survey items, specifically to identify and describe pressing organizational and operational stressors experienced broadly across PSP groups.

### 1.1. Occupational Spaces: Organizational and Operational Stressors

Duxbury and Higgins [16] broke down occupational stressors, stressors inherent to any employment, into Organizational (job context) and Operational (job content) stressors. Organizational stressors (job context) constitute one of the most common and tangible forms of occupational stress impacting PSP and have undeniable physiological and cognitive implications [16,17,18,20]. The management structure/philosophy of a PSP organization, and the resulting policy and practices, can constitute daily stressors at work [21]. The daily stressors might be mitigated or reduced with supportive and inclusive organizational structures, policies, and practices [22]; however, PSP work environments tend to be laced with inherent challenges including those tied to the para-militaristic organizational framework. For example, correctional work involves a hierarchical, rank-informed, relationship between different staff and the administration, and that organizational structure arguably facilitates stress, job dissatisfaction, and even burnout [23,24,25,26]. Maslach et al. [27] defined burnout as a “psychological syndrome in response to chronic interpersonal stressors on the job” developing when individuals are unable to cope successfully with the demands their employment places on themselves. Similarly, in a systematic review of stress and burnout among correctional officers, Finney and her colleagues [26] (p. 3) defined stress as “the psychological strain or distress resulting from exposure to unusual or demanding situations, known as stressors” [see also: 30]. There is similar evidence regarding organizational stress and burnout from other para-military structures, such as police [20,22], correctional [28], and paramedic services [29].

Organizational stress can also manifest as employees perceive a perpetual need to prove their value to their organization, which can facilitate perfectionism [30], retention or promotion insecurity [31], and unstable social supports [32,33,34]. The potential outcomes include occupational insecurity and compromised employee well-being. At a more granular level, a significant source of stress may emerge from how an organization facilitates the creation and maintenance of relationships between superiors and subordinates. Employees who perceive a lack of support between co-workers and leaders also describe experiencing significant stress [35].

Operational stress appears inherent to the PSP work context [16,17,18,20]. Operational stressors include exposures to frequent and chronic PPTEs which can impact diverse aspects of well-being [6,12,36]. For example, vocationally-related physical attacks or injuries, witnessing, being involved in, or delivering death notifications, and high-risk activities (e.g., high speed chases) are all operational stressors that can compromise mental health and well-being [6,37]. The operational realities can be exacerbated by fatigue, understaffing, and shift work [12,38,39,40,41,42]. Researchers have also evidenced the substantial negative impacts of shift work on employee performance [43]. For example, irregular working hours, overtime, and rotating shifts alter circadian rhythms, which can lead to sleep disruptions [32] and increased PTSD vulnerability [44]. Nevertheless, shift work is unlikely to disappear, which underscores the need for realistic sustainable solutions for individual PSP well-being [45].

### 1.2. Current Study

Occupational stressors, both organizational and operational, directly and indirectly impact employee occupational experiences and well-being. Relative to the general population, PSP report elevated exposures to organizational and operational stressors that contribute to acute and chronic deterioration of PSP mental health and well-being [6,46,47]. Researchers have also evidenced PPTEs as significant operational stressors for PSP [6,48]. Carleton and colleagues [14] quantitatively assessed for differences in operational and organizational stressors across PSP groups, finding the highest rated stressors were consistent across PSP groups; specifically, primary stressors included fatigue, finding time to stay in good physical condition, occupation-related health issues, not enough time to spend with family and friends, negative comments from the public, eating healthy at work, and PPTE exposures [14]. All stressors were associated with increased odds of screening positive for one or more mental disorders, suggesting several potential opportunities for possibly improving mental health by modifying organizational stressors; however, the impact of such experiences remains insufficiently understood. In our current study, we build on the work of Carleton and colleagues [14] by unpacking the sources and contexts of the organizational and operational stressors self-reported by PSP. We analyze what PSP report when asked to further comment in open-ended response fields. The results provide a more comprehensive understanding of how PSP experience occupational stress, and the stressors that are unique or not unique to PSP occupations. Moreover, enhancing understanding of the impact of occupational stressors on PSP can inform how organizations manage said stressors perhaps resulting in novel and potentially more effective processes that further support PSP recruitment, retention, and performance [48].

## 2. Methods

Following established guidelines for web-based surveys [49], we collected the current data using a web-based self-report survey made available to PSP participants from 1 September 2016 to 31 January 2017 in English or French. The survey was part of a larger study looking at the prevalence of mental disorders among PSP (for information on the greater study and survey design, see [4,16,46]). Participation was solicited through emails sent to actively working PSP, including civilian members working for police and volunteer firefighters. Recruitment of PSP was primarily through diverse public safety organizations (e.g., unions, organizations) but also involved social media. Emails informing PSP about the study were circulated by employers, professional associations, and researchers; including through institutional listservs. Of note, the former federal Minister of Public Safety and Emergency Preparedness, Mr. Ralph Goodale, assisted with informing potential PSP about the study through a public service announcement (i.e., video) that was created and disseminated.

The current study drew on PSP who provided qualitative responses to an open-ended item, “Do you have any other comments or concerns regarding work-related stressors?” within a section exploring stresses experienced at work (see Table 1). We complemented these responses with data provided by PSP who discussed organizational or operational stressors when asked to provide final comments regarding the survey through a second open-ended item, “If you have any additional information you would like to provide or additional feedback, please feel free to do so below” (see Table 2). Participants were placed into one of six PSP categories: correctional workers, federal police (Royal Canadian Mounted Police [RCMP]), firefighters, municipal/provincial police, paramedics, and public safety communications officials. The University of Regina Institutional Research Ethics Board (File #2016-107) provided ethical approval for the data collection.

We employed a semi-grounded constructed qualitative data analysis process that was thematically inductive and based on the identification and categorization of emergent themes from the data [50,51,52,53]. We exported the raw online survey data from Qualtrics into an archive file, and then imported the raw data into NVivo Pro 2019. We autocoded data from each respondent as a unique case, allowing us to assign demographic data (e.g., gender, occupation, race) to respondents as NVivo “attributes” which served as demographic variables during analyses. We coded respondents’ qualitative responses into ‘parent’ nodes, each representing a primary emergent theme, and we broke down each parent node into ‘child nodes’ representing subthemes within the greater parent node. Researchers became fully immersed in the data set as they coded [53]. First, we thematically organized 100 responses to create our codebook or coding scheme. Second, we coded the full dataset; repeatedly reviewing and axially coding our data and further developing the codebook until themes were exhaustive and mutually exclusive. Each excerpt including in the results is with permission from the participant, anonymized, and edited for grammar and spelling.

## 3. Results and Discussion

A total of 828 PSP (14% of total survey respondents) responded to the first open-ended item, “If you have any additional information you would like to provide or additional feedback, please feel free to do so below.” An additional 1,238 PSP (21% of total survey respondents) provided a response to the second open-ended question, “Do you have any other comments or concerns regarding work-related stressors?” Table 1; Table 2 show a breakdown of our study respondents by PSP occupation, including socio-demographic details.

From our respondents’ data seven themes related to occupational stressors emerged, including four organizational and three operational stressors. For each theme we provide contextual information before presenting excerpts from the data to demonstrate each finding. We then discuss each emergent theme. We focus first on specific organizational stressors consistently volunteered by our sample of PSP when asked about their occupational experiences: interpersonal work relationship dynamics; workload distribution; lack of resources; and administrative obligations. We then focus on operational stressors, other than PPTEs, that underpin our PSP participants’ work experiences: vigilance; work location; and interacting with the public.

### 3.1. Organizational Stressors (Job Context)

#### Interpersonal Work Relationship Dynamics

Supervisors, Management, and Administration. The ways employees feel they are treated by their colleagues, including their superiors, can negatively or positively shape experiences at work. For example, concerns arise during instances where employees feel supervisors or upper management treat them poorly, unfairly, or negatively, particularly in instances where relationships of power underpin the perceived treatment (e.g., feeling undervalued by a manager). Employees may then feel undermined, disrespected, or devalued. For example:

“My primary source of stress is not from traumatic incidents—but how I am supervised, micromanaged and berated during them. I have a supervisor who makes constant and fictitious complaints against other persons and as a result numerous employees have quit and moved elsewhere.”

(Participant 1271, male, municipal policer officer)

“Only certain rules apply to certain people and when you are away and someone is supposed to cover your duties doesn’t, you return to work with everything piled on your desk. But when the person that was supposed to cover your position is away you have to go do that job before your own. So when your work falls behind you get yelled at or written up for not doing your duties according to the requirements of the position. Then if you complain that the Manager is required to make the person do your duties when you are away they say you are not taking responsibility for not doing your job.”

(Participant 943, female, federal correctional officer)

“It also appears as though upper management is on a “witch hunt” to chastise and blame the membership any chance they get versus assessing situations and applying the appropriate remedies/decisions and standing by them. There is little confidence in the organization that the front line members are supported or “backed” to do the challenging job that present obvious risks and challenges where situations may not have unfolded the way it could have but where that is the “cost of doing business”, it is accepted and supported. The reality is the membership feels as though they will be hung out to dry if an action even resembles a mistake and no relief in pay/benefits/HR issues.”

(Participant 2710, male, RCMP officer)

Our participants’ words show how supervisors can create new layers of stress by promoting an atmosphere of perceived unfairness, as well as harassment (as evidenced in the words of participant 1271), that employees have to navigate. The presented sample quotes exemplify a feeling of a negative atmosphere created by supervisory colleagues, through perceptions of “fictitious complaints” (participant 1271), unequal treatment of employees by supervisors (Participant 943), or a perception of general mistreatment of all employees by administration (Participant 2710). Seemingly unjust methods of supervision, participant 1271 explained, not only directly create a source of stress for PSP, but also create tangential issues (e.g., self-terminations or transfers), demonstrating the scope of impact poor supervision tactics bear on self and work environments. Such practices are also evidenced in participant 2710′s expression of how workers internalize an organizational culture that places little confidence in senior management and consequently suggests PSP should prepare to expect blame for every decision and (in) action.

Colleagues. PSP across sectors indicated they have experienced or witnessed bullying and/or harassment among colleagues and staff in their workplace:

My [colleague] likes to bully people in and out of positions. If certain people do not like you they deliberately do things to put stress and pressure on you so that you will withdraw from positions.”

(Participant 943, female, federal Correctional Officer)

“Is easy to overlook the all too common issue of staff who exist in these roles who… have malicious intent or personal agendas. These people exist as predators in the workplace, undermining the value and achievements of others, and existing only to exalt themselves onto a pedestal above others out of an apparent need to gain approval as a better person. Unfortunately, these people are rotten to the core, and will go to unknown lengths to further their agendas causing completely unnecessary grief and turmoil to the careers and lives of their supposed peers.”

(Participant 4122, male, Paramedicine)

Our participants’ words indicate perceptions of intentional and strategic bullying by coworkers “in and out of positions” (participant 943) in their workplaces, all of which places undue and unnecessary additional stress on colleagues attempting to manage their workload. Such a strategy of specific PSP “causing completely unnecessary grief and turmoil” (participant 4122) to their colleagues creates new variants of organizational stress, such as the existence of “predators in the workplace” (Participant 4122), a stressor completely independent of PSP occupational demands. As evidenced in the words of Participant 4122, the interpretation of certain workers as having “malicious intent or personal agendas” which serve only to reaffirm their positioning in the workspace while devaluing others, adds pressure onto PSP trying to manage their own workloads.

Participant 4122 raises concerns over some PSP feeling their “days as a front line responder are numbered”. For some respondents, like participant 4122, bullying and harassment are ingrained so deep into the workplace culture that they feel the only avenue for relief is to transfer out of their workplace or quit their jobs altogether. Moreover, such interpretations negatively influence how PSP perceive their workplace and even their occupational duties and responsibilities. Speaking to the effects of workplace harassment on self, a PSP noted:

“Workplace harassment that occurred from [date removed] has left me a different person. It has impacted me severely, it’s like it has altered my brain to the point that I have never felt the same since it began. Seeing extreme trauma has been bad enough, but the harassment has, I feel, changed the way in which I process things. I feel as though my brain has been on overdrive ever since.”

(Participant 8892, female, RCMP officer)

As evinced in the words of participant 8892, workplace harassment can become psychologically traumatic. Participant 8892 reports harassment as having permanently “altered” their brain; the subculture of harassment diffused through their workplace has left them a different person. In sum, the words of our participants evidence a variant of workplace bullying, not only from those who observed such behaviors, but also from those who were victims.

### 3.2. Workload Distribution

#### Resources

Human Resources and Staffing. Participants expressed being affected by the lack of available human resources, they described working conditions that included understaffing, the unavailability of backfill for PSP on leave, and vacant positions. For example, a municipal officer noted:

“With less resources, the burnout rate is increased. This is exacerbated by guilt when needing to take time off for stress injuries and increases stigma.”

(Participant 1461, male)

Two PSP further commented:

“The demands of the job with little or no resources to address existing and new issues create challenges in getting ahead. There is little to no movement to address staffing shortages, or to make valuable or usable changes to make the job easier or more efficient.”

(Participant 2710, male, RCMP officer)

“Long term but unaddressed staffing shortages are hurting me very badly, both physically and mentally. I am working harder than I should, i.e., when compared to paramedic services elsewhere in the province, and I’m working longer than I should, i.e., epidemic of overtime. There is no relief in sight, and I’m very worried about my prospects of a healthy retirement.”

(Participant 5686, male, paramedic)

Across PSP sectors, participants consistently felt that having fewer resources negatively affects the effectiveness and efficiency of the labour force, evidenced, for example, among PSP who require days off feeling ashamed or guilty for needing them (participant 1461). Participant 5686 (paramedic) further notes how staffing shortages evoke feelings of compromised mental and physical well-being and increase already excessive workloads. In effect, a lack of resources can be deleterious to future career goals, such as the “prospects of a healthy retirement” (participant 5686).

Material Resources. Some PSP perceived the funding directed toward agencies of public safety as inadequate and, in consequence, compromising the capacity of PSPs to fulfill their general public safety obligations. For example:

“There is little actual concern from Management over … having adequate resources to have a safe workplace for first responders.”

(Participant 2497, male, RCMP officer)

“The main stressors all seem to be related and a result of no money in the department I work for which means: poor/old equipment (delay in replacing equipment or told to wait until next year), cutting positions, loosing bodies/back up, no money for overtime so we end up working short.”

(Participant 3613, male, RCMP officer)

Across both excerpts, the “constant pressure to perform more with ever shrinking support and resources” (Participant 1453, Male, Municipal Police) is echoed by the participants. In effect, financial or funding decisions manifest as intradepartmental and departmental challenges such as disparities between required equipment and available funding for equipment. The words of participant 3613 reveal how underfunding can result in improper equipment, or staffing relief, which leaves members working “short” (e.g., responding to calls understaffed) posing a risk to staff and public safety. Overall, the organizational stressor of budgeting limitations creates stress, challenges PSP capacities to effectively complete occupational tasks, and impedes the safety of PSP as they fulfill their occupational responsibilities.

Administrative obligations, specifically paperwork, emerged as a significant organizational stressor among PSP. Respondents identified that the quantity of paperwork added to the difficulties PSP already endure as they try to keep up with their workload. For example:

“The increased expectation that the organization has on us is getting overwhelming. We are expected to answer e-mails/phone calls/texts when off duty...when we do anything we are expected to be able to document every step we take whether it is a conversation with a co-worker, an investigation, or an administrative task…”

(Participant 6411, male, municipal police officer)

Participant 6411 indicates challenges originating from organizational stressors overall and administrative obligations specifically. The perception that the job is prioritized over other aspects of their lives (e.g., answering calls and so on while off shift) appears to further compound work-related stress by creating implicit expectations about needing to document every action. Many PSP echoed that the amount of required documentation was a key stressor and imposition on their workloads and time. Paperwork as a stressor was further intensified by a perception that organizations were continuously adding to the burden in various ways, such as new required documentation. Participant 2693, a male police officer, explained:

“The service continues to find new ways to add to the paperwork and complexity of what we do usually in the name of “capturing stats” but it all adds more time and more headaches on top of what the courts add to our caseload.”

(Participant 2693, male, municipal police officer)

Increased paperwork tied to practices viewed as unnecessary (i.e., “in the name of “capturing stats”, participant 2693) consumes PSP time and energy, adds complexity and volume to overloaded workers, and therein increases organizational stress. PSP describe the latent consequence of even more paperwork resulting from the expectation that one must compensate for others who are off duty without additional resources. Such practices increase workloads and distract PSP from completing tasks and responsibilities:

“Amount of paperwork is very excessive and demanding for files. When members go off duty sick it puts high burden on those left working because most of the time their shift is not filled due to financial restraints or no one willing to do overtime.”

(Participant 2877, female, RCMP officer)

Participant 2877 describes the lack of backfill of staff on leave as a burden for remaining staff driven in part by the excessive resulting paperwork and organizational demands.

### 3.3. Operational Stressors (Job Content)

#### Vigilance

The potentiality of risk often characterizes PSP work. Operational duties such as a call for service in the community or in a prison (e.g., as a police or correctional officer), and many other duties unique to PSP (e.g., putting out fires, responding to car accidents, answering calls for help) require a degree of vigilance uncommon in other occupations. On-shift PSP must always be ready to respond and to maintain public safety, with a limited ability to take on-shift downtime. The need for vigilance does not cease with the end of the workday. Respondents indicated a feeling of vigilance and always being “on” even while no longer on shift:

“Feeling like my job is never done. I go home, but on call for any crisis to arise, or can’t go out on my free time with my friends, or family because I fear for [their] safety in the public if I am identified by someone that dislikes me because of my career, or when I am at work, I won’t be home to protect my family if someone comes to fulfill one of the countless threats I receive.”

(Participant 6382, male, correctional services)

“Constant stressors—no relief—even when away on holidays there is still a level of stress below the surface. That level of stress can quickly jump out depending on conversations, other people’s actions or other simple reasons.”

(Participant 6644, male, RCMP officer)

Participant 6382 describes their operational responsibilities as continuing past shift end, meaning there is no down time and they are always on call. The respondents both describe operational behaviours, such as safety vigilance, as permeating their home life. Participant 6382 reports the necessity of vigilance given there is also the possibility to be “identified by someone that dislikes me because of my career”. Work permeates time off with family and friends because PSP have seen first hand the constant risk in which the public live (e.g., accidents) and thus remain vigilant off duty to secure the safety of those around them.

The safety and security of loved ones is also concerning for PSP when on duty, where they “won’t be home to protect” them (participant 6382). The participants described omnipresent concerns around safety, a “level of stress below the surface” (participant 6644) that is only exasperated by the feeling that their work “is never done” (participant 6382). PSP may be embroiled within risk potentiality informed by their work that creates a degree of stress that “can jump out depending on conversations, other people’s actions or other simple reasons” (participant 6644). Essentially, disentangling work from personal life becomes complicated as each informs the other, often with “no relief” (participant 6644), a byproduct being omnipresent vigilance. Participants explain how operational stress includes and extends beyond the work day.

### 3.4. Work Location

The geographic location of PSP work also informed its operational realities and the associated stressors. For example, PSP working in rural areas experience specific operational challenges due to the remote nature of their work location (e.g., rural towns, northern communities) and often vast geographical space they respond to calls for service within (see, for example [54]). PSP may be required to cover extensive geographic regions and are likely unfamiliar with the multitude of small communities within their region until after deployment. Indeed, rurality is shaped by a cultural context of familiarity (i.e., citizens know one another) and a sense of community and belonging [54,55,56]. Yet as participant 1550, a female EMT paramedic, indicates building rapport is challenging when they are “cycled through multiple towns on a regular basis”, both with community members and with other PSP. Accordingly, PSP lack the community-based knowledge that emerges when working in a consistent space and can inform operations. Moreover, being PSP within small rural communities can create a sense of isolation. For instance, Participant 3001, a male RCMP officer, spoke of multiple operational stressors: the isolation they endure working alone; the additional stress of responding to calls for service alone and without the needed resources to manage the situation; and always “being on call”. PSP in such situations lack the support that comes with working with a partner and the added safety of responding to calls for service with at least one other person.

### 3.5. Interacting with the Public

#### 3.5.1. Public Information

PSP described how the public interprets the diverse public safety professions—and the roles of those within the fields—as a source of operational stress affecting PSP when on or off duty. Some attributed challenging interactions with citizens, like participant 1187, to the compromising (mis)information the media and television programming provide which informs public perceptions. Such portrayals can negatively affect the public image of PSP and reduce the degree of understanding among the public of the unique challenges inherent to their occupational work:

“There is a perception I have come across in the public that I believe stems from movies and TV as much as from news outlets, that the people in my job are incompetent and/or thugs. Every time you have to explain to someone that you are just trying to get though a day that they themselves couldn’t understand is tiring and mentally exhausting.”

(Participant 1187, male, correctional officer)

Participant 1187 describes new challenges that may arise when interacting with the public who are unaware of the nuances of the PSP role. Participant 1187 explain how even when just “trying to get through a day” (Participant 1187), PSP are readily facing possible scrutiny because the public may be interpreting their actions through a rather negative lens (e.g., that they are “incompetent and/or thugs”, Participant 1187). As such, time is spent explaining how their actions are tied to their occupational responsibilities; a new layer of stress in an already “mentally exhausting” work content.

#### 3.5.2. Public Harassment

Compounding such experiences are, as respondents indicate, harmful and discouraging behaviors from the public toward PSP which can be psychologically draining and hurtful:

“I am often abused by the public who seek our help. Though not physically, there is a lot of verbal abuse. I have been called awful names, yelled at, endured racist and sexist comments (as a guy), screamed at and being spoken to rudely. Also, people often threaten me with complaints that they don’t go through with forcing me to tell my supervisor who then tells me to complete paperwork as a result. We still get people who believe the ambulance can provide magical drug services or that we can suddenly jump the line for a stubbed toe versus someone who is in cardiac arrest… Patient care is fine. It’s the public behavior that brings me the greatest stress…”

(Participant 1988, Male, Paramedic)

Participant 1988, a paramedic, reported on the harsh comments from the public received when performing his occupational duties in an efficient and appropriate manner; abuse suffered while serving his community. The paramedic also reveals how public reactions, particularly negative reactions to PSP, is a source of stress, and for some PSP “the greatest stress” (participant 1988); making a long term career in public safety feel unbearable on self. Stressors include the public refusing to cooperate and uncertainty around how the public will react when PSP arrive on site, which only increases the risk faced when operational. PSP are to balance public safety (e.g., care, control, response) with public behaviour, navigating a fine line between responding to needs while simultaneously mitigating public scrutiny.

#### 3.5.3. Public Scrutiny

PSP are responsible for implementing new policies and practices, some successful and some less so, yet feel public scrutiny over their actions intensifies when complications arise or calls for service do not proceed optimally. PSP feel quickly blamed despite their best efforts.

*“It’s hard to nail down one or more particular issues on a given day. Police work is constantly changing… Members are under relentless pressure from the communities, their supervisors, their co-workers, and their families. Everything needs to be perfect every time and if it’s not, society and the organization are looking to blame someone. The problem is that we, as Canadian society are constantly looking to place blame, sometimes first responders do their best and it’s just a bad decision or simply didn’t work.”*

(Participant 9, male, RCMP officer)

*“Politics and special interest groups are influencing the way police do their job. When the fallout occurs because of bad policy or legislation, the very people who initiate it or enact it, blame the very people who warned against it. This constant public blaming is creating unimaginable stressors to the job.”*

(Participant 464, male, municipal police officer)

*“Police officers are treated like numbers and what counts the most is what the public thinks. We are guilty until proven innocent. The public opinion of police officers has dramatically changed over the last few years, increasing the level of stress of officers. Everywhere we go, we get filmed, yelled at and accused of misconduct. Our hands are tied when it comes to do our work and years after years we lose the ability to do our jobs…”*

(Participant 1332, male, municipal police officer)

Not every operational decision and action will result in optimal outcomes; nevertheless, some PSP appear to feel vilified by the public despite their best intentions and efforts. The words of participants 464 and 9 reveal how operational stress emerges in part because of the blame the public places on PSP despite the lack of agency of PSP in their need to adhere to policies—including the very policies they may have cautioned against implementing. Political decisions certainly impact PSP occupations, but PSP can feel blamed by society when policy fails to have the intended impact. Public perception and blameworthiness have the potential to seep into the workplace culture and, as participant 1332 suggests, manifest into a negative work environment across PSP ranks.

#### 3.5.4. Public Education

PSP recommend public education to curb such negativity while increasing respect and empathy the public direct toward PSP work:

“The public does not understand what police officers go through. I wish they were better informed about specifically what traumatic events and stress that officers go through, not just that some suffer from PTSD. I think if the public was better informed it would facilitate a better relationship with the police… “

(Participant 9204, male, municipal police officer)

As evidenced above, Participant 9204 believes better informing the public about the nuances of PSP work may inform public scrutiny, improve relationships, and reduce unnecessary occupational stress among PSP.

## 4. Conclusions

Extant data, predominantly aggregated by PSP occupation, increasingly support occupational stressors (composed of organizational and operational stressors) as contributing to poor mental health for PSP. However, the way individual PSP experience occupational stressors and there associated impacts remain unclear. Further, while some forms of operational stresses (e.g., exposure to potentially psychologically traumatic events) are arguably unavoidable to PSP, other occupational stressors may exist that can be mitigated, improving the overall mental health of our PSP. The open-ended responses provided by our PSP participants clarify individual experiences of organizational and operational stressors, and highlight potentially unknown occupational stressors that are shared across all PSP occupations. For instance, respondents consistently mentioned a lack of human resources and a lack of material resources as stressors in their line of work; noting how their job context—the organization of responsibilities and coworkers—shape the work environment. As such, organizational stressors experienced by various PSP occupations appear largely related to hierarchical (e.g., with supervisors) and interpersonal (e.g., with colleagues) interactions at work that can manifest as perceptions of unfair workload distributions (e.g., supporting new, untrained colleagues), and can be amplified by perceptions of diminishing resources and constantly increasing administrative obligations. Indeed, our current results build on previous work by elucidating PSP perceptions of organizational stressors causing occupational challenges significantly more frequently than operational stressors.

PSP from our study commonly reported substantial difficulties with many diverse organizational stressors (i.e., interpersonal relationships, workload distribution issues, resources, and administrative obligations). Here, interpersonal work relationships were a significant source of stress, not only from colleagues and coworkers, but also based on interactions with supervisors, management, and administration. Relationships, for example, were strained by feelings of frustration rooted in perceptions of co-worker competency, work ethic, and workload distribution. Unsurprisingly, our respondents commonly expressed a perceived lack of human resources, resulting in situations where PSP were not available to backfill PSP on leave thus positions remaining vacant, long shifts, or overtime requirements, which can all evoke feelings of compromised well-being. Further, perceptions of inadequate material resources, such as reduced funding or insufficient access to required equipment, create a perception of risk to PSP safety and a reduction in effectiveness. Compounding resource limitations was an expressed perceived increase in administrative duties, with a significant emphasis on an already high paperwork load that is constantly increasing, sometimes for reasons that are not considered by PSP to be necessary (e.g., ”capturing stats”).

PSP respondents also emphasized the impact of common operational stressors not directly related to PPTE exposures. Specifically, our respondents expressed a pressure to remain vigilant both on the job and while off duty (‘i.e., always on’). The source of the pressure varied from needing to protect one’s family or friends to being prepared to be called into work at any time. Our respondents also noted the impact of work location, particularly work in remote or rural areas that could lead to feelings of isolation and always being on call, but also how the familiarity of rural communities in some cases was a positive influencer on stress. Finally, our respondents clearly noted how interactions with the general public were at times quite stressful, particularly when dealing with public misinformation and negativity toward PSP occupations. Participants also reported experiencing intense public scrutiny and a responsibility to provide education to the public about their occupational responsibilities.

Considering the feedback taken from our participants, we propose several recommendations to support efforts intended to reduce the impact of occupational stressors. First, organizations’ policies and protocols could be assessed to promote a positive atmosphere of respect, fairness, and employee appreciation. Key here is understanding how interpersonal work relationships (and the associated stressors) are shaped by interactions and relationships with supervisors, management, and administration as well as colleagues and coworkers. Second, organizations could assure bullying and harassment policies in the workplace not only exist, but are effective and accessible, and as necessary, employers could promote training on workplace bullying and harassment. Third, perceptions of uneven workload distributions commonly exist among PSP occupations, to rectify said perceptions, PSP organizations could strive toward greater transparency, fairness, and efficiency in workload scheduling and distribution. Fourth, training practices could be reviewed to ensure training is provided to commensurate to occupational requirements, and that sufficient staff are available to account for shortages thus easing the workload burden experienced by PSP. Fifth, organizational policies could reflect the perceived need for material resources expressed by PSP, and either provide the missing resources or clearly and transparently communicate the restrictions impacting the provision of needed resources. Seventh, organizations could consider improving (e.g., streamlining or centralizing) administrative responsibilities to reduce onerous paperwork, or work together with active PSP to develop mutually acceptable processes.

In response to operational stressors, organizations could consider the impact of PSP occupations on personal life, with a particular focus on perceptions of safety and responsibility while off-duty, and in response provide tools for employees to help accommodate their off duty concerns. Moreover, organizations’ could consider the nuances of work locations (e.g., the benefits versus disadvantages of working in rural communities) when developing policies and procedures. Finally, organizations could consider methods of improving interactions between PSP and the general public (e.g., media campaigns to counteract negative portrayals of PSP), promoting a framework for more positive relationships between PSP and the public; relationships with the potential to enhance public trust and confidence in PSP work in the future. PPTE exposures are necessarily a part of PSP work; however, our results demonstrate a novel contribution in suggesting reducing or mitigating those operational stressors that can be reduced may produce significant improvements in PSP mental health and well-being.

### Limitations and Future Work

Organizational stressors are not unique to PSP occupations; however, the PSP in our sample appeared to disproportionately report challenges with organizational stressors relative to operational stressors. Our respondents may have emphasized organizational stressors as a result of our open-ended questions following predominantly operational (i.e., PPTE) scale items, which may have biased respondents to focus on other stressors. In the future, we recommend explicitly exploring organizational and operational stressors independently.

The disproportionate representation of organizational over operational stressors in our data does not diminish the emphasis our PSP placed on operational stressors (i.e., stressors related to job content). For PSP, operational stressors related to PPTE exposures at work are associated with occupational stress and mental health challenges [6]. The comments from our respondents complement and extend the current conceptualization of PSP operational stressors beyond a focus on the significance of PPTEs in PSP work [14].

We caution that our sample was self-selected and from a population of Canadian PSP, suggesting that synthesis of our data and results with a broader sample of national and international PSP is necessary for broad representation, recognizing that, as with all qualitative data, generalizability is limited. Indeed, self-selection bias, a limitation of the current study, suggests that the comments made by our participants may not be representative of all Canadian PSP. However, the fact that theme saturation emerged across variables and public safety professions provides comfort about the applicability and accuracy of the findings as well as the nature of the comments being representative of respondent experiences. Moreover, given our study was designed to identify common stressors across public safety occupations—rather than within occupations—we suggest future researchers examine the stressors unique to each PSP occupation (e.g., how do the stressors experienced by police officers different than those experienced by paramedics?)

Overall, our qualitative results build upon previous quantitative research that underscored the problematic impact of organizational and operational stressors on PSP. Our results showcase the depth and breadth of stressors that can and do negatively impact the mental health of PSP, perhaps unexpectedly highlighting how colleagues, superiors, organizations, and communities can be directly injurious or can exacerbate injuries from PPTE exposures. The identified mechanisms of injury may also offer attainable opportunities for PSP to make changes towards better supporting their shared mental health by changing how they interact with each other and with systems. Our results also suggest that the individual health and well-being of PSP can be improved through more traditional approaches utilized in other non-PSP organizations. Future researchers should include assessments of the impacts and interactions of familial stressors and individual difference variables on occupational stressors and PSP mental health.

## Figures and Tables

**Table 1:** Demographics of the 1238 individuals who responded to the question “Do you have any other comments or concerns regarding work-related stressors?”

Regional Employment	*%* (*n*)
Western	51.9 (642)
Ontario	28.7 (355)
Atlantic/Eastern/Northern	12.8 (158)
Quebec	5.7 (71)
Other	0.9 (12)
**Sex**	
Male	65.2 (807)
Female	34.3 (424)
**Age**	
19–29	3.8 (47)
30–39	22.7 (281)
40–49	35.4 (438)
50–59	31.9 (395)
60 and older	5.8 (72)
**Most Common Occupations**	
RCMP	26.6 (329)
Municipal Police Officer	17.3 (214)
CSC, Operational (Institutional)	10.3 (127)
Firefighter	9.5 (117)
Paramedic/ACP	7.2 (89)
Paramedic/PCP	4.8 (59)

**Table 2:** Demographics of the 828 individuals who responded to the question “If you have any additional information you would like to provide or additional feedback, please feel free to do so below”.

Provincial Employment	*%* (*n*)
Western	52.1 (431)
Ontario	29.6 (245)
Atlantic/Eastern/Northern	12.1 (100)
Quebec	5.8 (48)
Other	0.5 (4)
**Sex**	
Male	67.1 (556)
Female	32.5 (269)
**Age**	
19–29	5.3 (44)
30–39	21.9 (181)
40–49	38.0 (315)
50–59	28.7 (238)
60 and older	5.8 (48)
**Most Common Occupations**	
RCMP	25.1 (208)
Municipal Police Officer	17.1 (142)
CSC, Operational (Institutional)	8.7 (72)
Firefighter	12.4 (103)
Paramedic/ACP	8.1 (67)
Paramedic/PCP	5.9 (49)
